# Symptom Clusters Using the Edmonton Symptom Assessment System in Patients With Bone Metastases: A Reanalysis Comparing Different Statistical Methods

**DOI:** 10.4021/wjon403w

**Published:** 2012-02-19

**Authors:** Luluel Khan, Gemma Cramarossa, Emily Chen, Janet Nguyen, Liying Zhang, May Tsao, Cyril Danjoux, Elizabeth Barnes, Arjun Sahgal, Lori Holden, Flo Jon, Shaelyn Culleton, Edward Chow

**Affiliations:** a Rapid Response Radiotherapy Program, Odette Cancer Centre, Sunnybrook Health Sciences Centre, University of Toronto, Canada

**Keywords:** Symptom cluster, Bone metastases, Statistical analysis, Edmonton symptom assessment system, Palliative radiotherapy.

## Abstract

**Background:**

To determine whether symptom clusters in patients with bone metastases vary when extracted using three different statistical methods. To compare the temporal composition of symptom clusters in responders versus non-responders to palliative radiation treatment.

**Methods:**

A previous dataset of 518 bone metastases patients who completed the Edmonton Symptom Assessment System (ESAS) was used in this study. Clusters derived using Principal Component Analysis (PCA) in our previous study were compared to symptom clusters extracted using Hierarchical Cluster Analysis (HCA) and Exploratory Factor Analysis (EFA). Clusters were derived at baseline, and 1, 2, 4, 8 and 12 weeks after radiation treatment. The patient sample was further divided into responders versus non-responders to radiotherapy. The three statistical methods were performed to identify clusters in the subgroups at each time point.

**Results:**

A complete consensus between HCA, EFA and PCA for the number and composition of symptom clusters was not reached at any time point. Furthermore, little correlation in clusters was found between the three statistical methods despite the use of an identical data set. As expected, different symptom clusters were observed in the responders and non-responders with all three statistical methods. In addition, clusters varied at each time point within each subgroup. Depression and anxiety were consistently found in the same cluster.

**Conclusion:**

The quantity, composition, and occurrence of symptom clusters varied based on which statistical method was employed. The use of a common analytical method is necessary for consistency and comparison purposes in future symptom cluster research.

## Introduction

Bone metastases are a common manifestation in advanced cancer patients. Approximately 65 - 75% of prostate and breast cancer patients as well as 30 - 40% of lung cancer patients develop bone metastases [1]. Complications can include substantial pain, and increased risk of spinal cord compression, hypercalcemia, and fractures. Patients with bone metastases often experience multiple symptoms that simultaneously influence their quality of life. Previous studies have discovered the tendency of two or more related symptoms to consistently occur in conjunction, forming symptom clusters [2, 3].

Symptom cluster research in oncology is a useful tool to improve symptom management [4]. However, if inconsistencies due to the employment of several statistical methods exist, a true set of validated symptom clusters cannot be identified. Our previous study employed Principal Component Analysis (PCA) to extract symptom clusters in data collected from patients with bone metastases using the Edmonton Symptom Assessment System (ESAS) [5]. Our present study derived symptom clusters using two additional commonly used statistical methods, Hierarchical Cluster Analysis (HCA) and Exploratory Factor Analysis (EFA) [6]. All methods employed effectively extract clusters of related symptoms. Analysis of symptom clusters was also conducted in two subgroups: responders and non-responders to radiation treatment. The primary aim of this study was to determine whether the implementation of different statistical methods derive significantly different clusters. The secondary objective was to compare symptom clusters in responders and non-responders over time using the three statistical methods.

## Patients and Methods

This study utilized the same dataset as our previous study. ESAS questionnaires were administered during routine clinical assessment at baseline, and at 1, 2, 4, 8 and 12 months post-radiation treatment. The ESAS is an 11-point scale that assesses nine symptoms, where zero indicates the absence of a symptom and 10 indicates the strongest presence of a symptom. The symptoms measured are: pain, fatigue, nausea, depression, anxiety, drowsiness, appetite, sense of well-being and shortness of breath. ‘Pain’ was specific to bone pain at the irradiated site for the purposes of this study. This questionnaire has been corroborated in cancer patients. Patient demographics, cancer history, disease status and analgesic consumption were recorded at the first visit. Demographics included age, gender, inpatient or outpatient status, weight loss of greater than 10% over the previous six months and Karnofsky Performance Status (KPS).

Questionnaires and data were collected by a trained research assistant in person at baseline and via telephone interview at all follow-ups. Verbal consent was obtained from all patients. The dataset compiled from 518 bone metastases patients undergoing palliative radiation treatment was identical to that used in our previous study to avoid inconsistencies such as varied sample populations or different assessment tools. Ethics approval was obtained from Sunnybrook Health Sciences Centre. The entire process was consistent with the principles set by the Declaration of Helsinki on conducting clinical research.

### Statistical analysis

PCA, EFA and HCA were employed in the present study to identify symptom clusters.

PCA with varimax rotation was performed on the nine ESAS items to identify any interrelationships between symptoms at each follow-up time point. This statistical method transforms a number of observed variables (symptoms) into a smaller number of variables (called principal components). Each principle component is a cluster. The highest loading factor score predicted the assignment of individual symptoms to a cluster. Significant clusters were determined by an Eigenvalue greater than 0.8 and each component explained more than 10% of the variance. Internal consistency and reliability of clusters was measured using Cronbach’s alpha.

EFA is the most commonly used analytical method in oncology symptom cluster research. EFA is unique because it assumes symptoms in a cluster are correlated by latent factors, which bind the symptoms together [7, 8, 9]. The maximum likelihood method and the *varimax* orthogonal rotation method were utilized for approximately multivariate normal data to assess covariance between symptoms. The number of factors (clusters) was determined based on an eigenvalue higher than 0.8, which indicates that almost 10% of variance in the symptom is shared with the latent factor after controlling for the correlation between factors. Together these two methods identify and finalize the items (symptoms) that belong to each cluster. The PROC FACTOR procedure in Statistical Analysis Software (SAS version 9.2) was conducted for this analysis. The Cronbach’s alpha was calculated to measure the internal consistency of the clusters.

HCA identifies symptom clusters using average linkage between groups. This method focuses on classification by grouping similar symptoms in a cluster and separating clusters from each other. The PROC VARCLUS runs clusters on the basis of *centroid* components. The R^2^ values of each symptom with its own cluster and with its nearest cluster were calculated. The 1-R^2^ ratio is the ratio of one minus the value in the “Own Cluster” column to one minus the value in the “Next Closest” column. The lower the ratio, the more separated the clusters are. HCA produces a dendrogram, which is a visual representation of clusters. Symptoms that converge earlier on the dendrogram are more closely related than those that those that converge later.

### Criteria for responders and non-responders

Patients were divided into two subgroups based on their pain response to radiation and analgesic consumption in order to compare symptom cluster composition in responders and non-responders. A responder was defined as a patient with complete (CR) or partial response (PR). CR implies a pain score of 0 at the irradiated site with no increase in analgesic uptake. PR was described as a decrease in the patient’s worst pain score of at least 2 at the treated site without analgesic increase, or an analgesic reduction of at least 25% from baseline without pain increase. Patients who did not indicate a complete or partial response were considered non-responders. Response definitions were based on those set by the International Bone Metastases Consensus [10]. The three statistical methods were used to derive symptom clusters for both subgroups.

## Results

### Symptom clusters at baseline

Patient characteristics are summarized in Table 1.

**Table 1:** Patient Characteristics

Characteristics	N (%)
Age at radiation (year)	
Mean ± SD	67.9 ± 10.9
Median (range)	68 (31 - 93)
Sex	
Male	280 (54%)
Female	238 (46%)
Weight loss ≥ 10% in the past 6 months	
No	261 (50%)
Yes	180 (35%)
Unknown	77 (15%)
Karnofsky Performance Status	
Mean ± SD	61.2 ± 14.1
Median (range)	60 (10 - 100)
Total Morphine Equivalent	
Mean ± SD	103 ± 234
Median (range)	30 (0 - 3600)
Primary cancer sites	
Breast	127 (25%)
Prostate	117 (23%)
Lung	130 (25%)
GI	39 (8%)
Unknown	34 (7%)
Others	71 (14%)

SD: standard deviation.

The PCA statistical approach was previously performed with varimax rotation on the nine ESAS symptoms. At baseline, there were three symptom clusters identified. Cluster 1 consisted of fatigue, drowsiness, pain. Cluster 2 consisted of depression and anxiety, and Cluster 3 included nausea, appetite and shortness of breath [5].

**Figure 1:**
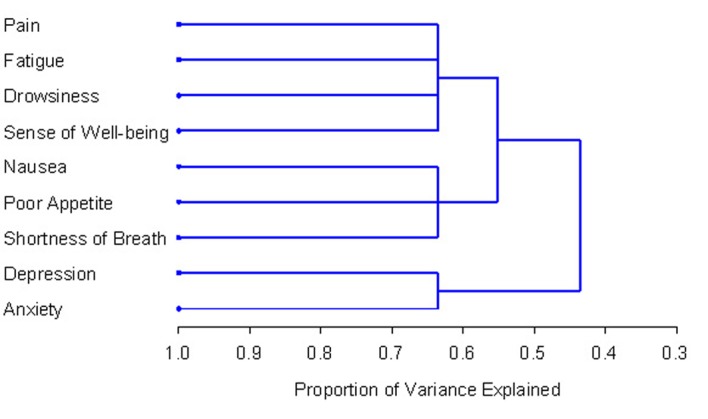
PROC TREE procedure generated dendrogram displaying three cluster solution and cluster hierarchy. More similar symptoms were joined together earlier.

The HCA method revealed identical clusters to PCA. The centroid cluster algorithm split the nine symptoms into two clusters (Table 2). Cluster 1 contained pain, fatigue, nausea, drowsiness, poor appetite, sense of well-being and dyspnea. Cluster 2 included depression, and anxiety. The first cluster explained 47% of the total variation and the second cluster explained 84%. In accordance with this statistical method, Cluster 1 was further divided into two 3-item clusters because it had the smaller proportion of variation (Table 3). The dendrogram in Figure 1 provides a visual representation of the clusters. A one-cluster solution explained 43.6% of total variation, and two and three cluster solutions explained 55.2% and 63.7%, respectively.

**Table 2:** Initial Two Symptom Clusters Identified Using Centroid Cluster Algorithm in HCA

		*R*^2^		
		Own Cluster	Next Cluster	1 – *R*^2^*_own cluster_*
				1 – *R*^2^*_own cluster_*
Cluster 1	Pain	0.3863	0.0663	0.6573
	Fatigue	0.5332	0.1531	0.5512
	Nausea	0.4157	0.1303	0.6719
	Drowsiness	0.5374	0.1420	0.5392
	Poor Appetite	0.5290	0.1088	0.5285
	Sense of Well-being	0.5762	0.2457	0.5618
	Shortness of Breath	0.3387	0.0558	0.7004
Cluster 2	Depression	0.8383	0.2745	0.2228
	Anxiety	0.8383	0.1686	0.1944

HCA: Hierarchical Component Analysis; First Cluster explained 47%, second Cluster explained 84% of the total variation. The Cluster 1 will be split due to the smallest proportion of variation (47%).

**Table 3:** Final Two Symptom Clusters Determined Using HCA With Centroid Cluster Algorithm

		*R*^2^		
		Own Cluster	Next Cluster	1 – *R*^2^*_own cluster_*
				1 – *R*^2^*_own cluster_*
Cluster 1	Pain	0.4842	0.1412	0.6006
	Fatigue	0.6378	0.2182	0.4633
	Drowsiness	0.6305	0.2299	0.4798
	Sense of Well-being	0.6007	0.3140	0.5820
Cluster 2	Depression	0.8383	0.2525	0.2163
	Anxiety	0.8383	0.1655	0.1937
Cluster 3	Nausea	0.5776	0.2012	0.5288
	Poor Appetite	0.6176	0.3123	0.5561
	Shortness of Breath	0.5129	0.1464	0.5706

HCA: Hierarchical Component Analysis. One Cluster 1 explained 44%, two Clusters explained 55%, and three Clusters explained 64% of the total variation

The nine ESAS items were also analyzed using EFA, which divided the symptoms into two clusters with eigenvalue > 1.0 and proportion > 10% (Table 4). Cluster 1 consisted of drowsiness, fatigue, sense of well-being, appetite, pain, nausea, and dyspnea. Cluster 2 included depression and anxiety, and was similar to one of the clusters present using PCA and HCA. The internal consistency of each cluster was greater than 0.8, indicative of strong internal consistency. The first cluster explained 82.5% of the total variance, and the second cluster explained the remaining 17.5% of the total variance. Table 5 displays the clusters determined using EFA.

**Table 4:** Eigenvalues and Proportions of Variance for the Nine ESAS Items Using EFA

Component	Eigenvalue	Proportion	Cumulative
1	10.7297	0.8254	0.8254
2	2.2704	0.1746	1.0000
3	0.4049	0.0311	1.0311
4	0.0902	0.0069	1.0381
5	0.0228	0.0018	1.0398
6	0.0027	0.0002	1.0400
7	-0.0714	-0.0055	1.0346
8	-0.1698	-0.0131	1.0215
9	-0.2794	-0.0215	1.0000

BPI: Brief Pain Inventory; EFA: Exploratory Factor Analysis. From eigenvalues and proportions of variance, two factors (clusters) were retained (eigenvalue > 1.0 and proportion > 10%), and the cumulative variance showed up to 100%.

**Table 5:** Factor Loadings and Final Communality Determined Using EFA

	Factor 1	Factor 2	Final communality
Drowsiness	0.67	0.20	0.49
Fatigue	0.66	0.23	0.49
Sense of Well-being	0.65	0.36	0.55
Poor Appetite	0.63	0.20	0.44
Pain	0.51	0.14	0.28
Nausea	0.49	0.26	0.31
Dyspnea	0.44	0.13	0.21
Depression	0.29	0.89	0.87
Anxiety	0.23	0.69	0.53
% of variance	82.5%	17.5%	
Cronbach’s alpha	0.82	0.81	

EFA: Exploratory Factor Analysis.

### Symptom clusters over time

PCA, EFA and HCA were also applied to data collected at follow-up points. The cluster results at baseline and at 1, 2, 4, 8 and 12 weeks post-treatment are displayed in Table 6.

**Table 6:** Symptom Clusters Identified at Baseline and Follow-Ups Using Three Statistical Methods

Symptom	Baseline (n = 518)			1 week FU (n = 272)			2 week FU (n = 297)			4 week FU (n = 266)			8 week FU (n = 231)			12 week FU (n = 193)		
	PCA	EFA	HCA	PCA	EFA	HCA	PCA	EFA	HCA	PCA	EFA	HCA	PCA	EFA	HCA	PCA	EFA	HCA
Depression	Δ	Δ	Δ	Δ	Δ	Δ	Δ	Δ	Δ	Δ	Δ	Δ	Δ	—	Δ	Δ	Δ	Δ
Anxiety	Δ	Δ	Δ	Δ	Δ	Δ	Δ	Δ	Δ	Δ	Δ	Δ	Δ	—	Δ	Δ	Δ	Δ
Fatigue	Ο	Ο	Ο	Ο	Ο	Ο	O	Ο	Ο	Ο	Ο	Ο	Δ	—	Ο	Ο	Ο	Ο
Drowsiness	Ο	Ο	Ο	Ο	Ο	Ο	O	Ο	Ο	Ο	Ο	Ο	Ο	—	Ο	Ο	Ο	Ο
Pain	O	Ο	O	X	Ο	X	O	Ο	Ο	Δ	Ο	O	Δ	—	Δ	Ο	Ο	Ο
Nausea	X	Ο	X	X	Δ	X	Δ	Δ	Δ	Ο	Ο	Ο	Ο	—	Ο	X	Ο	X
Poor appetite	X	Ο	X	X	Ο	X	Δ	Ο	Δ	Ο	Ο	Ο	Δ	—	Δ	Δ	Ο	Δ
Dyspnea	X	Ο	X	O	Ο	Ο	—	Ο	—	—	Δ	—	—	—	—	X	Ο	X
Poor well-being	O	Ο	O	Δ	Δ	Δ	Δ	Δ	Δ	Δ	Δ	Δ	Δ	—	Δ	Δ	Δ	Δ

FU: follow-up; PCA: Principal Component Analysis; EFA: Exploratory Factor Analysis; HCA: Hierarchical Component Analysis. Symptoms with corresponding symbols indicate they were in the same cluster. Dash indicates the symptom was not present in any clusters.

At one week following palliative radiation treatment (n = 272), PCA and HCA identified identical system clusters. Cluster 1 was composed of depression, anxiety and well-being. Cluster 2 included fatigue, drowsiness and dyspnea, and Cluster 3 consisted of pain, nausea and poor appetite. EFA identified two clusters: Cluster 1 which included depression, anxiety, nausea, and well-being and Cluster 2 which consisted of fatigue, drowsiness, pain, poor appetite, and dyspnea.

At the two week follow-up (n = 297), all three methods identified two clusters. Once again PCA and HCA derived identical clusters. Cluster 1 included depression, anxiety, nausea, poor appetite, and well-being. Cluster 2 consisted of fatigue, drowsiness and pain. EFA’s two clusters were composed of fatigue, drowsiness, pain, poor appetite, and dyspnea; and depression, anxiety, nausea and sense of well-being.

At the 4 week follow-up (n = 266), all three statistical methods identified similar clusters. EFA and HCA both distinguished a cluster composed of fatigue, drowsiness, pain, nausea, and poor appetite. EFA identified one other cluster, which included depression, anxiety, dyspnea, and well-being. HCA’s remaining cluster was similar, but excluded dyspnea. PCA identified a similar cluster to the one derived by both EFA and HCA, excluding pain. PCA’s second cluster included depression, anxiety, pain, and well-being.

At 8 weeks following radiation treatment (n = 231), EFA did not identify any symptom clusters. PCA identified two clusters, one composed of drowsiness and nausea, and the other of depression, anxiety, fatigue, pain, poor appetite, and well-being. Similar clusters were identified through HCA. Cluster 1 using HCA included fatigue, drowsiness, and nausea. Cluster 2 consisted of depression, anxiety, pain, poor appetite, and well-being.

At the 12 week follow-up (n = 193), PCA and HCA identified three identical clusters. Cluster 1 was composed of depression, anxiety, poor appetite, and well-being. Cluster 2 included fatigue, drowsiness and pain. Cluster 3 consisted of nausea and dyspnea. EFA identified two clusters of the nine ESAS symptoms. Depression, anxiety and well-being were present in Cluster 1. Cluster 2 was comprised of fatigue, drowsiness, pain, nausea, poor appetite, and dyspnea.

### Consistency of symptom clusters findings using PCA, EFA and HCA

As expected, there were variations in both the number and composition of symptom clusters identified using the three statistical methods. Nonetheless, some symptoms, such as depression and anxiety, consistently occurred together within the same cluster despite the statistical method employed. Poor well-being clustered with these two symptoms at every follow-up, but not at baseline. In addition, fatigue and drowsiness frequently occurred in conjunction with each other.

### Symptom clusters in responders versus non-responders over time

Our previous study reported the development of symptom clusters in both responders and non-responders to palliative radiation treatment using PCA. Our current study extracted clusters in these two subgroups using EFA and HCA. PCA identified variation in the number and composition of clusters in the responders group over time. Three clusters were present at baseline, one week and one month. Two clusters were observed at two weeks, two months and three months. Three clusters were consistently observed in non-responders, except for week eight at which only two clusters were identified. Comparison of the two groups reveals disparities in symptom clusters. Table 7 contains detailed results of PCA analysis in responders and non-responders.

**Table 7:** Symptom Clusters in Responders Versus Non-Responders Subgroups Using PCA

Method	Symptom	Baseline		1 week FU		2 week FU		4 week FU		8 week FU		12 week FU	
		NR n = 518	R n = 518	NR n = 140	R n = 132	NR n = 148	R n = 149	NR n = 132	R n = 134	NR n = 122	R n = 109	NR n = 95	R n = 98
	Depression	Δ	Δ	Δ	Δ	Δ	Δ	Δ	Δ	Δ	Δ	Δ	Δ
	Anxiety	Δ	Δ	Δ	Δ	Δ	Δ	Δ	Δ	Δ	Δ	Δ	Δ
	Fatigue	X	X	Ο	Ο	Ο	Δ	Ο	O	Ο	Δ	Ο	Δ
	Drowsiness	X	X	Ο	Ο	Ο	Δ	Ο	O	Ο	Δ	Ο	Δ
PCA	Pain	X	X	X	X	X	Δ	Δ	X	Δ	—	Ο	—
	Nausea	O	O	X	X	Δ	O	Ο	X	Ο	O	X	Δ
	Poor appetite	O	O	X	Ο	Δ	O	X	O	Δ	O	O	O
	Dyspnea	Ο	Ο	Ο	Ο	X	—	X	O	—	O	X	O
	Poor well-being	X	X	Δ	Δ	Δ	O	Δ	Δ	Δ	Δ	Δ	Δ

PCA: Principal Component Analysis; FU: follow-up; NR: non-responders; R: responders. Symptoms with corresponding symbols indicate they were in the same cluster.

Dash indicates the symptom was not present in any clusters.

Derivation of symptom clusters using EFA as presented in Table 8 revealed significant differences in symptom clustering between responders and non-responders. However, two symptom clusters were generally observed in both groups at most time points, with the exceptions of non-responders at one week, which included three clusters, and responders at eight weeks which did not demonstrate any clustering.

**Table 8:** Symptom Clusters in Responders Versus Non-Responders Subgroups Using EFA

Method	Symptom	Baseline		1 week FU			2 week FU		4 week FU		8 week FU		12 week FU	
		NR n = 518	R n = 518	NR n = 140	R n = 132	NR n = 148		R n = 149	NR n = 132	R n = 134	NR n = 122	R n = 109	NR n = 95	R n = 98
	Depression	Δ	Δ	Δ	Δ	Δ		Δ	Δ	Δ	Δ	—	Δ	Δ
	Anxiety	Δ	Δ	Δ	Δ	Δ		Δ	Δ	Δ	Δ	—	Δ	Δ
	Fatigue	Ο	Ο	Ο	Ο	Ο		Ο	Ο	Ο	Ο	—	Ο	Δ
	Drowsiness	Ο	Ο	Ο	Ο	Ο		Ο	Ο	Ο	Ο	—	Ο	O
EFA	Pain	Ο	Ο	X	Δ	Δ		Δ	O	Δ	Δ	—	Ο	Δ
	Nausea	Ο	Ο	X	Δ	Δ		Ο	O	Δ	Ο	—	Ο	O
	Poor appetite	Ο	Ο	X	Ο	Δ		Ο	Δ	O	Δ	—	Ο	Δ
	Dyspnea	Ο	Ο	Ο	Ο	Ο		Δ	Δ	Ο	O	—	Ο	Δ
	Poor well-being	Ο	Ο	Δ	Δ	Δ		Ο	Δ	Δ	Δ	—	—	Δ

EFA: Exploratory Factor Analysis; FU: follow-up; NR: non-responders; R: responders. Symptoms with corresponding symbols indicate they were in the same cluster. Dash indicates the symptom was not present in any clusters.

As indicated in Table 9, the use of the HCA method also revealed incongruities in symptom clusters over time within each subgroup and between subgroups. Three clusters were observed in both responders and non-responders at every time point, with the exception of week eight where only two clusters were noted.

**Table 9:** Symptom Clusters in Responders Versus Non-Responders Subgroups Using HCA

Method	Symptom	Baseline		1 week FU			2 week FU		4 week FU		8 week FU		12 week FU	
		NR n = 518	R n = 518	NR n = 140	R n = 132	NR n = 148		R n = 149	NR n = 132	R n = 134	NR n = 122	R n = 109	NR n = 95	R n = 98
	Depression	Δ	Δ	Δ	Δ	Δ		Δ	Δ	Δ	Δ	Δ	Δ	Δ
	Anxiety	Δ	Δ	Δ	Δ	Δ		Δ	Δ	Δ	Δ	Δ	Δ	Δ
	Fatigue	X	X	Ο	Ο	Ο		Δ	Ο	Ο	Δ	Δ	Ο	Δ
	Drowsiness	X	X	Ο	Ο	Ο		Δ	Ο	Ο	Δ	Δ	Ο	Ο
HCA	Pain	X	X	X	X	X		Δ	Δ	X	O	—	Ο	X
	Nausea	O	O	X	X	Δ		X	Ο	X	Δ	O	X	O
	Poor appetite	O	O	X	O	—		X	Δ	Ο	O	Δ	O	Ο
	Dyspnea	Ο	Ο	Ο	Ο	X		—	—	O	—	O	X	X
	Poor well-being	X	X	Δ	Δ	Δ		X	Δ	Δ	O	Δ	Δ	Δ

HCA: Hierarchical Component Analysis; FU: follow-up; NR: non-responders; R: responders. Symptoms with corresponding symbols indicate they were in the same cluster. Dash indicates the symptom was not present in any clusters.

The three statistical methods collectively revealed incongruent temporal trends in responders and non-responders. In addition, all three methods identify a variation in clustering from baseline in both responders and non-responders.

## Discussion

It is our understanding that this is the first longitudinal study in patients with bone metastases receiving radiation treatment that compares symptom clusters derived by different statistical methods. As in the original study, the patients were also divided into responder and non-responder subgroups. The current study utilized the same data set as our previous study in order to minimize extraneous factors such as sample population and assessment tools, both of which affect symptom cluster results. Symptom clusters were analyzed using PCA, EFA, and HCA for each subgroup at baseline and at identical follow-up time points. PCA detects clusters by minimizing variables into a smaller number of components [11]. EFA takes into account the covariance between symptoms [7]. HCA clusters variables of similar quantitative patterns.

No strong correlations were revealed between the clusters derived by the three statistical analyses. EFA identified the least amount of clusters, evident by the presence of only two clusters at most follow-up time points and the absence of clustering at week eight. HCA and PCA identified more symptom clusters, and clusters were seen at every follow-up point when these methods were employed. The disparity among the symptom clusters derived by each method is indicative of the influence of the statistical method on the quantity and composition symptom clusters.

There are a limited number of previous studies on symptom clusters in cancer patients that have used more than one statistical method; however these studies were primarily cross-sectional in nature. Maliski et al. [12] examined symptom clusters in prostate cancer patients using cluster analysis, factor analysis and Pearson correlations. Henoch et al. [13] employed the same three statistical methods to derive clusters in lung cancer patients. Gleason et al. [14] employed four different statistical methods to extract symptom clusters in brain tumour patients at two time points. Results of these studies indicated some comparable clusters when utilizing different methods; however they did not reveal a complete consensus.

A current concern is the lack of consensus as to which analytical method should be universally utilized to derive symptom clusters. PCA is the least favourable due to its disregard of the occurrence of errors [15]. EFA and HCA are thus more ideal methods since they take into account both co-occurrence and relatedness [11]. EFA also considers a fundamental factor that may be responsible for the symptom clustering. HCA is valuable for identifying subgroups of patients with similar symptom clustering, which may be useful for targeting specific interventions for a subgroup. The ideal method should be the most clinically meaningful, meaning that the clusters should occur frequently in patients and provide insights into further symptom management research [6].

The temporal stability of system clusters is advantageous for advancement in symptom management; however identical clusters are not expected to remain over time [16-18]. The presence of symptoms is generally fluctuating, as indicated by changes in the cluster quantity and composition in both responders and non-responders. All three statistical methods uncovered discrepancies among clusters at each follow-up time point. Kirkova and Walsh [19] proposed that at least 75% of the symptoms in a cluster, including the most prevalent symptom, must remain within the cluster over time in order for it to be considered stable. In our study, following these criteria, no symptom clusters are stable. Further studies investigating the development of symptom clusters over time are essential to improving symptom management.

The disparity between responder and non-responder symptom clusters is likely due to the reduction of pain following radiation treatment. The aggravation, improvement, or disappearance of a single symptom may subsequently alter cluster characteristics over time since symptoms in the same cluster are related. A decrease in pain medication for responders may also be a significant factor in the variance of symptom clusters between the subgroups over time, affecting both the incidence of pain as well as the side effects associated with analgesics. Among non-responders, symptom cluster instability may be caused by disease progression, modified interventions for lasting symptoms, or the long-term effects of various treatments.

The limitations of this study include the nature of both the assessment tool and the sample population. Cancer patients encounter a wide array of symptoms, thus the nine-item ESAS may not adequately assess the comprehensive range of symptoms each patient is experiencing. This negatively impacts system clustering as the presence of additional clusters may be observed upon the addition of more specific symptoms. Also, the primary cancer each patient has been diagnosed with varies, thus different patient populations may experience a slightly different range of symptoms.

The analysis of symptom clusters is a valuable resource for oncologists; however concerns that must be addressed include discrepancies caused by the various analytical methods. A consensus on one ideal statistical method will allow for relevant comparison among results of different studies. Currently, published studies have utilized several different methods of analysis which hinders the comparability of results. Inconsistencies in statistical method result in symptom cluster incongruities, as demonstrated by our findings. Clinicians’ ability to apply findings to potentially improve treatment and symptom management is thus impeded. A universal analytical method will provide a basis upon which studies on symptom clusters for patients with bone metastases can be compared to better understand their symptom experience.
